# Supplementary material for: Investigating lytic polysaccharide monooxygenase-assisted wood cell wall degradation with microsensors
Source: Nat Commun. 2022 Oct 21;13:6258. doi: 10.1038/s41467-022-33963-w (PMC9586961; doi:10.1038/s41467-022-33963-w)
Supplement: Supplementary file 1 — Supplementary Information [file 41467_2022_33963_MOESM1_ESM.pdf]

SUPPLEMENTARY INFORMATION FOR

Investigating Lytic Polysaccharide Monooxygenase-assisted wood cell wall degradation with microsensors

Hucheng Chang[1], Neus Gacias Amengual[1], Alexander Botz[1], Lorenz Schwaiger[1], Daniel Kracher[1,2], Stefan Scheiblbrandner[1], Florian Csarman[1], Roland Ludwig[1]*

[1] Department of Food Science and Technology, Institute of Food Technology, University of Natural Resources and Life Sciences, Vienna, Muthgasse 18, 1190 Vienna, Austria.

[2] Present Address: Institute of Molecular, Graz University of Technology, Petersgasse 14, 8010 Graz, Austria.

* Corresponding author: Roland Ludwig. Email: roland.ludwig@boku.ac.at

This file includes:

Supplementary Figures 1–8

Supplementary Tables 1–2

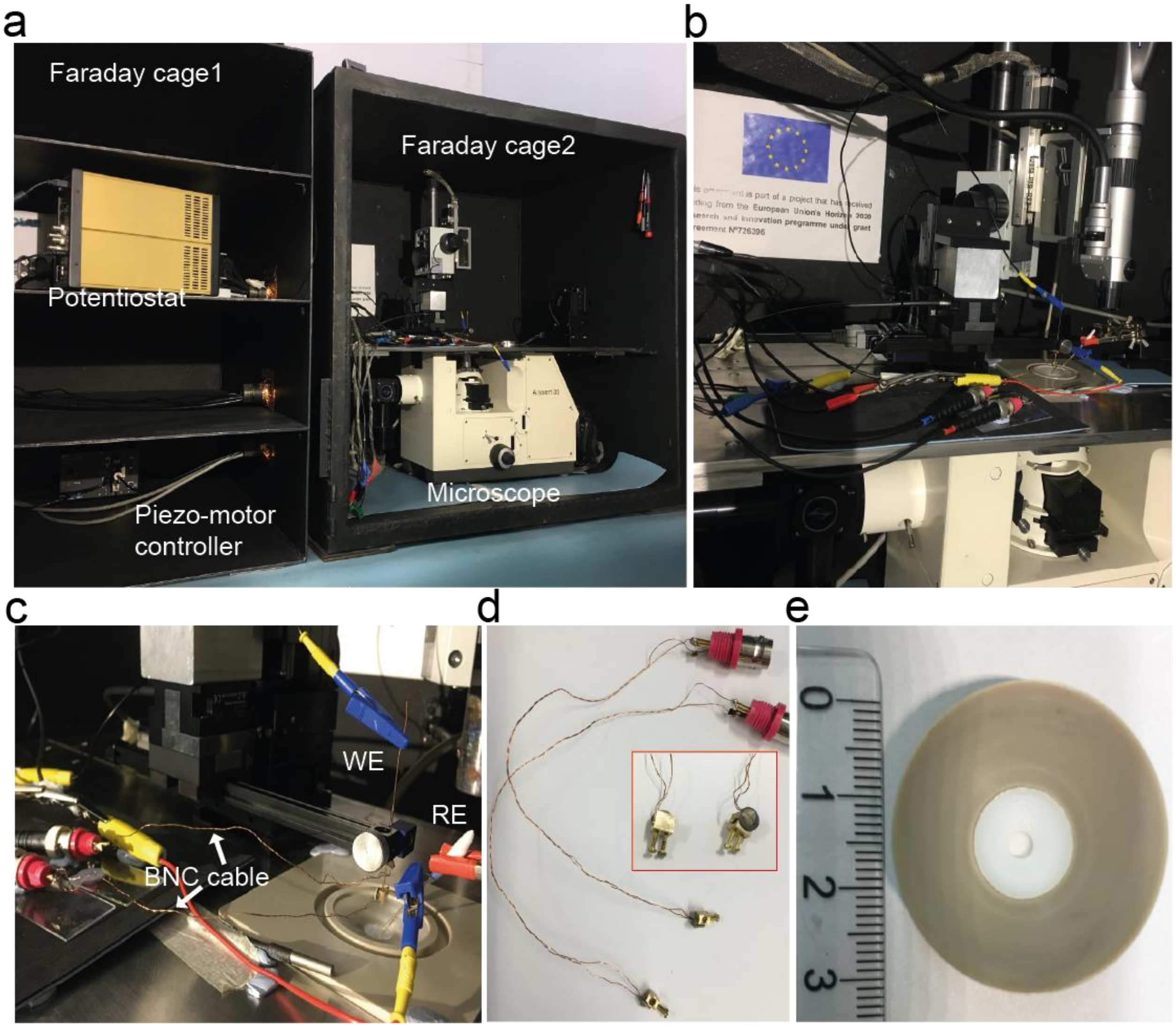

Supplementary Figure 1. The experimental setup of the SECM system. (a) A potentiostat and a micromanipulator controller placed in a Faraday cage. A micromanipulator, a sample holder (electrochemical cell), the top digital microscope and the inverted optical microscope are placed in the second Faraday cage. The micromanipulator is placed on the level stainless-steel board, and the circle sample holder is inlaid in the middle hole of this stainless-steel board. The distance **(b)** and close **(c)** view of the two-electrode electrochemical setup and the circle sample holder as an electrochemical cell. A micro(bio)sensor mounted on the cantilever of the micromanipulator is used as a working electrode, and a miniaturized Ag|AgCl is used as reference and counter electrode. **(d)** The combination of a piezo ceramic plate and a brass holder is connected with cables. **(e)** The circle sample holder (Ø: 32 mm) with an embedded Teflon ring (Ø$_{out}$: 12 mm, Ø$_{in}$: 3 mm,) serves as an electrochemical cell.

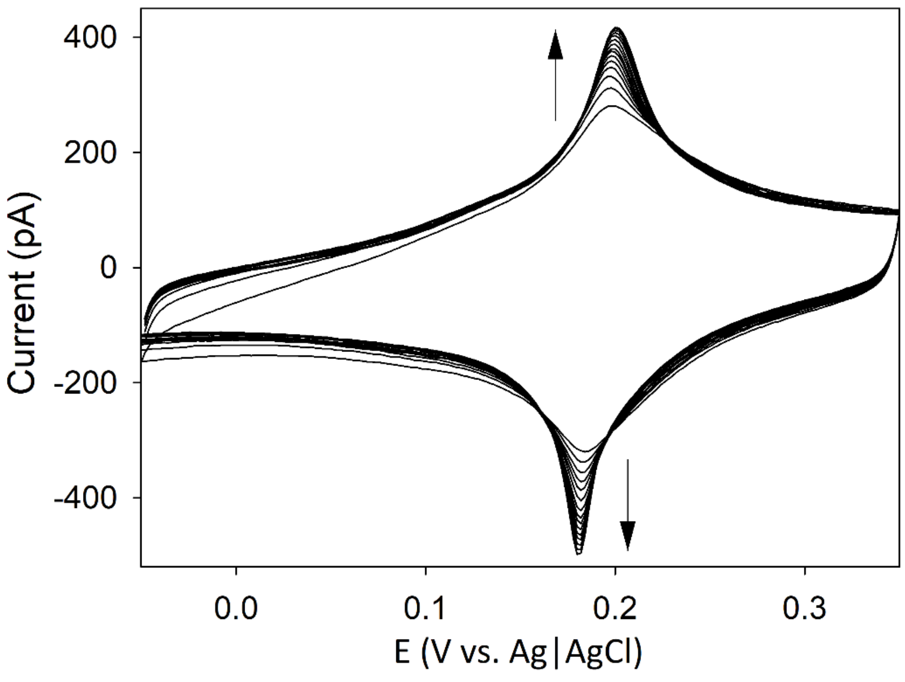

Supplementary Figure 2. Electrochemical activation of Prussian blue film. Cyclic voltammograms during electrochemical activation of the Prussian blue on a Pt ultramicroelectrode at a scan rate of 50 mV s^{-1} in 0.1 M HCl with 0.1 M KCl. The arrows showed the trend of current peak change with continued scanning (15 cycles). The scanning was started at -0.05 V.

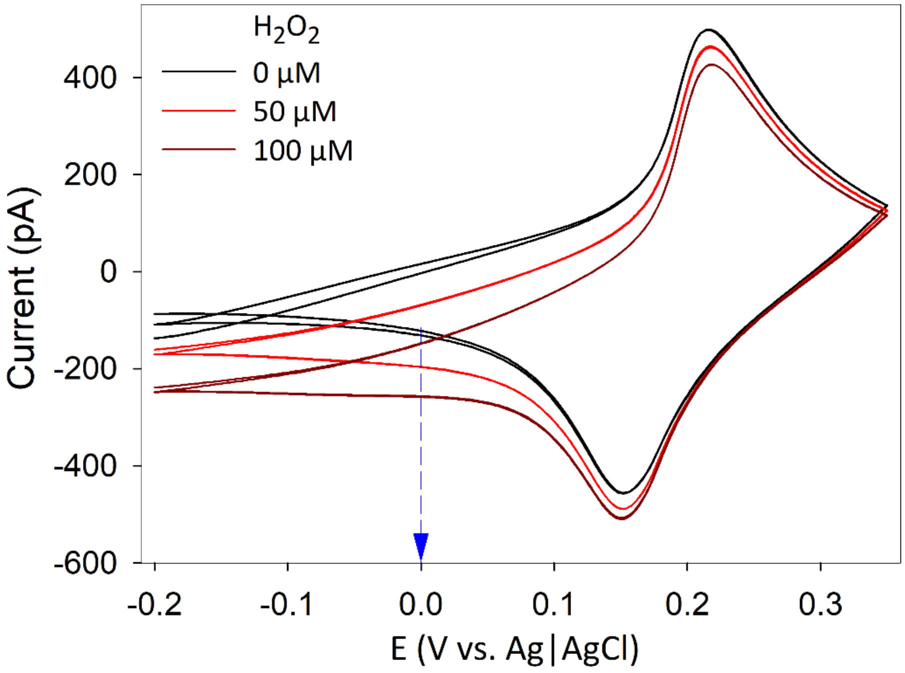

Supplementary Figure 3. Voltammetry characterization of an H_2O_2 microsensor. Cyclic voltammograms of a Prussian blue modified Pt ultramicroelectrode in 50 mM air-saturated acetate buffer, pH 5.5 in the absence (black line) and presence of 50 (red line) or 100 µM (dark red line) H_2O_2. The scan rate is 50 mV s^{-1}. The blue arrow indicates the potential (0.0 V) selected for all amperometric measurements using H_2O_2 microsensors.

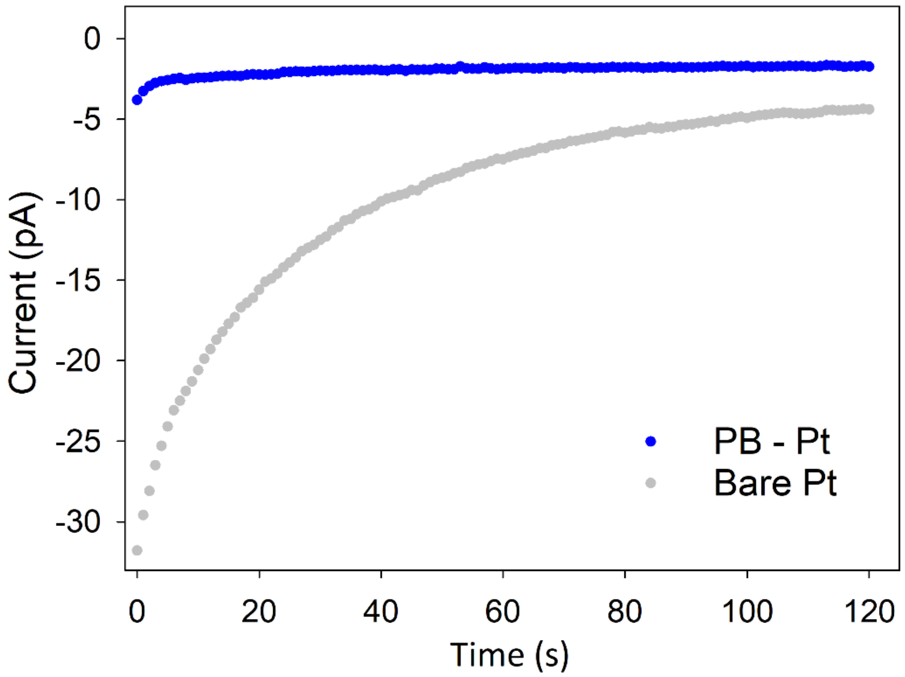

Supplementary Figure 4. Low activity of the Prussian blue for reducing O_2. Amperometric response of a Prussian blue modified (blue dots) and a bare (gray dots) Pt ultramicroelectrode in 50 mM air-saturated acetate buffer, pH 5.5 at an applied potential of 0.0 V vs. Ag|AgCl.

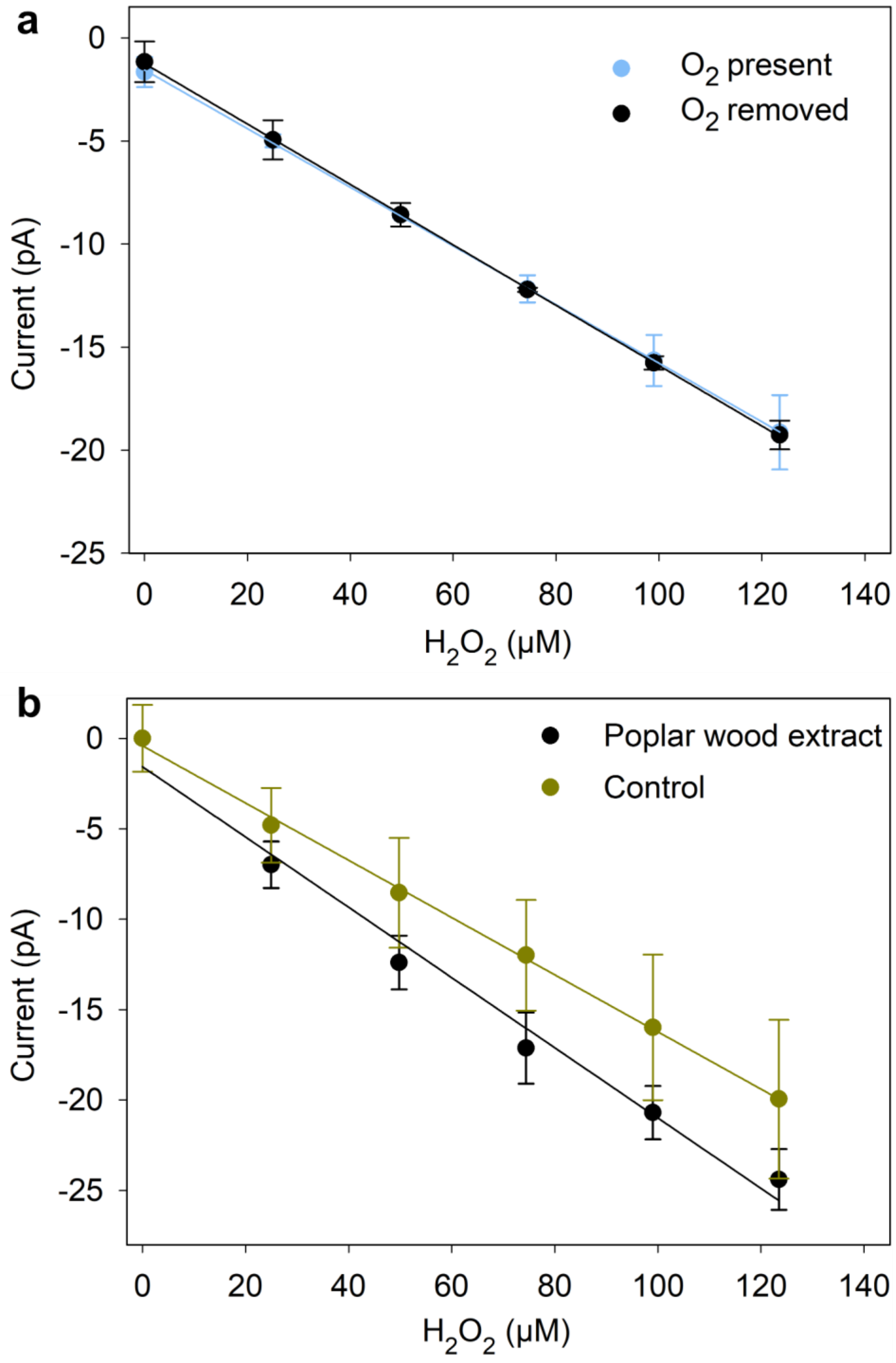

Supplementary Figure 5. Test the interference effect of O_2 and poplar wood extract on the H_2O_2 microsensors. a Calibration plots of H_2O_2 microsensors in 50 mM sodium acetate buffer, pH 5.5, in the presence (light blue: 0.142 µA µM^{-1}) and absence (black: 0.147 µA µM^{-1}) of O_2. **b** Calibration plots of H_2O_2 microsensors in 50 mM sodium acetate buffer, pH 5.5, in the presence (dark green: 0.158 µA µM^{-1}) and absence (black: 0.194 µA µM^{-1}) of poplar wood extract. Data in panels (a) and (b) are shown as mean values, and error bars show SD (n = 3, independent experiments). Extraction was performed for 16 h in ultrapure water at 22 °C using 20 % (w/w) freshly ground powder obtained from debarked poplar wood (particle size < 250 µm) and the solution was clarified by filtration prior to use.

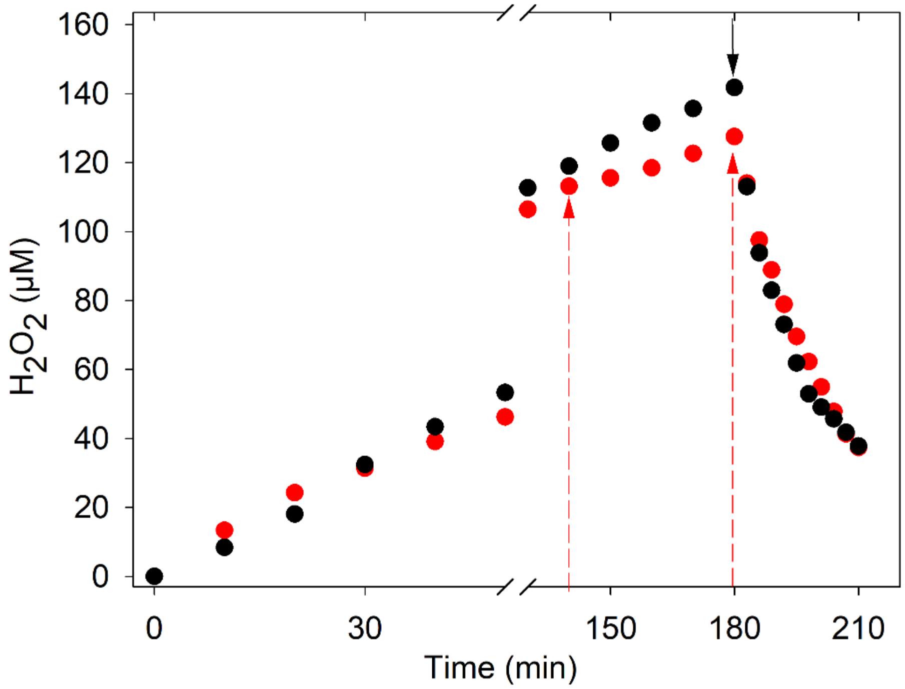

Supplementary Figure 6. The effect of reductant *N. crassa* CDHIIA on the localized H_2O_2 concentration. *C. hotsonii* CDH (1 μM) and 2 mg mL^{-1} cellobiohydrolases were applied for continual production of H_2O_2 during the whole time-course. The red arrows indicate the addition of LPMO and *Nc*CDHIIA in sequence, and the black arrow indicates the addition of LPMO and *Nc*CDHIIA together.

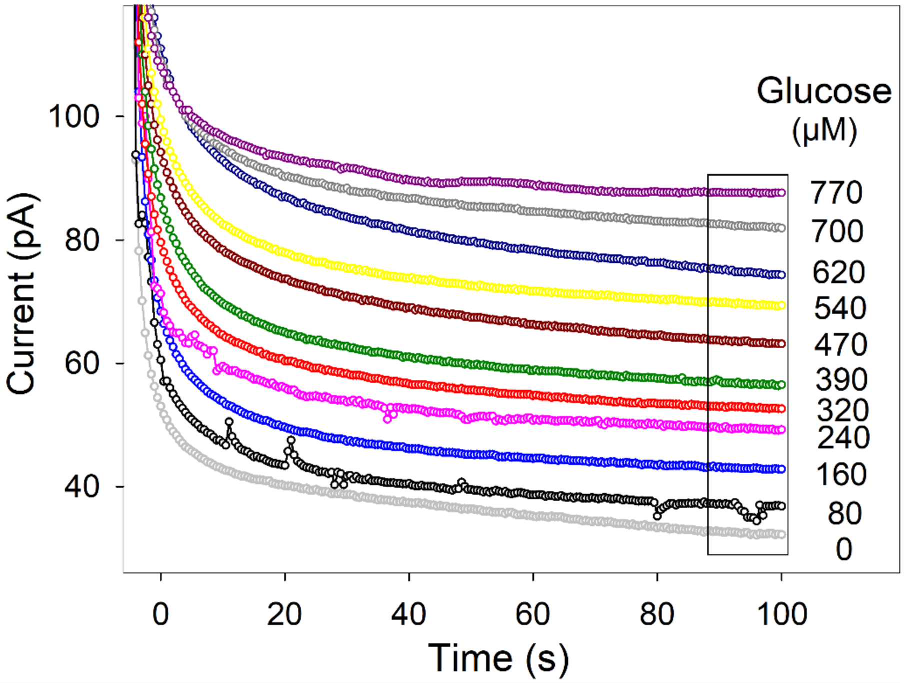

Supplementary Figure 7. Characterization of glucose microbiosensors. Amperometric response of a glucose microbiosensor to varying concentrations of glucose measured in 50 mM phosphate buffer solution of pH 6.0, at an applied potential of 0.55 V vs. Ag|AgCl.

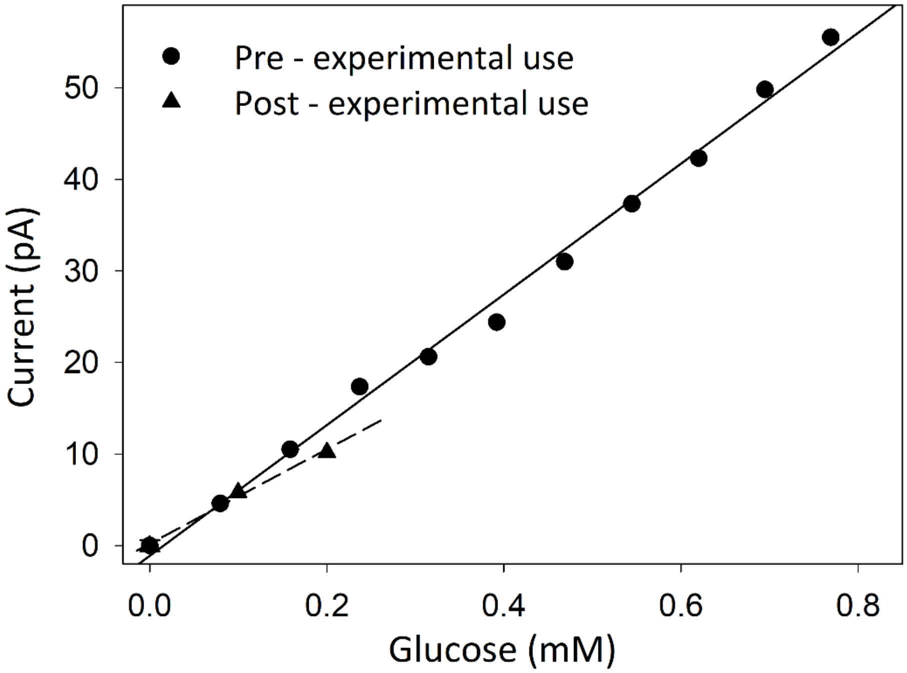

Supplementary Figure 8. Stability of glucose microbiosensors. Calibration plots of a glucose microbiosensor in Supplementary Figure 6 before (sensitivity: 71.4 pA mM^{-1}) and after (sensitivity: 51.0 pA mM^{-1}) 2 h of experimental use.

Supplementary Table 1. Analytical parameters of three independent H_2O_2 microsensors. The amperometric measurements were performed in 50 mM sodium acetate buffer, pH 5.5, at 20 °C in the presence of different concentrations of H_2O_2.

Parameter	Sensor 1	Sensor 2	Sensor 3	Average
Sensitivity [pA μM^{-1}]	0.093	0.088	0.083	**0.088** ± 0.005
Electrode diameter [µm]	1.13	1.27	1.22	**1.21** ± 0.07
Electrode area [μm^2]	1.00	1.26	1.17	**1.15** ± 0.13
Sensitivity [pA μM^{-1} μm^{-2}]	0.093	0.069	0.071	**0.078** ± 0.130
Noise [pA]	0.19	0.15	0.14	**0.16** ± 0.03
Limit of detection LOD [µM]	6.3	5.2	5.0	**5.5** ± 0.7
Limit of quantitation LOQ [µM]	21.0	17.3	16.7	**18.3** ± 2.3
Linear range [µM]	25–200	25–200	25–200	**25–200**
Correlation coefficient R^2	0.998	0.999	0.999	**-**

Supplementary Table 2. Analytical parameters of three independent glucose microbiosensors. The amperometric measurements were performed in 50 mM potassium phosphate buffer, pH 6.0, at 20 °C in the presence of different concentrations of glucose.

Parameter	Sensor 1	Sensor 2	Sensor 3	Average
Sensitivity [pA μM^{-1}]	0.064	0.045	0.043	**0.051** ± 0.012
Electrode diameter [μm]	1.50	1.42	1.56	**1.50** ± 0.07
Electrode area [μm^2]	1.77	1.58	1.91	**1.75** ± 0.16
Sensitivity [pA μM^{-1} μm^{-2}]	0.036	0.029	0.023	**0.029** ± 0.007
Noise [pA]	0.22	0.24	0.2	**0.22** ± 0.02
Limit of detection LOD [μM]	10.3	16.0	14.0	**13.4** ± 2.9
Limit of quantitation LOQ [μM]	34.3	53.3	46.7	**44.8** ± 9.6
Linear range [μM]	80–400	80–400	80–400	**80–400**
Correlation coefficient R^2	0.988	0.992	0.993	**-**
